# Home Water Treatment Habits and Effectiveness in a Rural Arizona Community

**DOI:** 10.3390/w7031217

**Published:** 2015-03-18

**Authors:** Nathan Lothrop, Sarah T. Wilkinson, Marc Verhougstraete, Anastasia Sugeng, Miranda M. Loh, Walter Klimecki, Paloma I. Beamer

**Affiliations:** 1 Environmental Health Sciences, Mel and Enid Zuckerman College of Public Health, The University of Arizona, 1295 N. Martin Avenue, Tucson, AZ 85724, USA; 2 Superfund Research Program, The University of Arizona, 1110 E. South Campus Dr., Tucson, AZ 85721, USA; 3 Institute of Occupational Medicine, Research Avenue North, Riccarton, Edinburgh, EH14 4AP; 4 Department of Pharmacology and Toxicology, College of Pharmacy, The University of Arizona, P.O. 210207, Tucson, AZ 85724, USA

**Keywords:** drinking water, Safe Drinking Water Act, reverse osmosis, rural health, arsenic

## Abstract

Drinking water quality in the United States (US) is among the safest in the world. However, many residents, often in rural areas, rely on unregulated private wells or small municipal utilities for water needs. These utilities may violate the Safe Drinking Water Act contaminant guidelines, often because they lack the required financial resources. Residents may use alternative water sources or install a home water treatment system. Despite increased home water treatment adoption, few studies have examined their use and effectiveness in the US. Our study addresses this knowledge gap by examining home water treatment in a rural Arizona community. Water samples were analyzed for metal(loid)s, and home treatment and demographic data were recorded in 31 homes. Approximately 42% of homes treated their water. Independent of source water quality, residents with higher income (OR = 1.25; 95%CI (1.00 – 1.64)) and education levels (OR = 1.49; 95%CI (1.12 – 2.12)) were more likely to treat their water. Some contaminant concentrations were effectively reduced with treatment, while some were not. We conclude that increased educational outreach on contaminant testing and treatment, especially to rural areas with endemic water contamination, would result in a greater public health impact while reducing rural health disparities.

## 1. Introduction

Under the Safe Drinking Water Act (SDWA) [1], the United States Environmental Protection Agency (USEPA) is authorized to set health-based guidelines for contaminants in drinking water. Implementation of these guidelines for public water systems is the responsibility of individual states and often falls on local municipalities. Very small municipal water suppliers (serving <500 people) are more than twice as likely to violate microbial and chemical contaminant guidelines compared to larger municipal utilities [2–4]. In states with more rural or decentralized populations, a larger fraction of the population is served by small providers. In the state of Arizona alone, there are 428 very small and 187 small (serving 500 - 3,300 people/system) municipal water providers [5]. Private wells serving fewer than 25 individuals are exempt from SDWA regulations altogether, and while some states suggest inspection and testing guidelines to private well owners [6], local governments have no legal jurisdiction over private well water quality. Thus, complying with drinking water guidelines is ultimately the responsibility of the individual well owners. In the United States (US), approximately 13 million unregulated, unmonitored private domestic water wells provide potable water to 43 million people (15% of the population) [2,7]. Considering increased contaminant guideline exceedances in smaller municipal water supplies and lack of regulation of contaminants in private well water, sizeable populations, often in rural areas, may be exposed to waterborne contaminants above health-based guidelines. This may put these populations at risk for potential long-term health outcomes, adding to rural health disparities [4].

In 2011, there were 7,170 USEPA Maximum Contaminant Level (MCL) exceedances by very small and small municipal providers, affecting 3,157 providers and 1,756,597 people, respectively [5]. In a national survey of 2,100 private wells, 23% contained >1 contaminant concentration greater than the MCL or another health-related benchmark, while 50% had elevated levels of contaminants affecting aesthetics (e.g. odor, taste or staining) [8]. Contaminants found at levels above guidelines were most often naturally occurring inorganic chemicals, such as metal(loid)s [8]. In areas with endemic high concentrations of natural contaminants (often inorganic chemicals), reaching compliance can be difficult for both municipal and in-home treatment systems using proven contaminant reduction technologies, as contaminant levels in untreated water may be too high to be reduced by treatment systems [2,3,9].

Nevertheless, US drinking water quality is among the safest in the world [10], afforded by treatments like rapid sand filtration, iron filtration, advanced oxidation processes, reverse osmosis, and chlorination [11]. These advanced technologies are most commonly used by municipal water suppliers serving multiple households [10], yet some are also available to individual homes as both point-of-entry (whole home) and point-of-use (at the tap) systems.

However, few studies have investigated water treatment habits and potential needs for targeted water treatment education of more vulnerable populations such as families with young children or pregnant women [12]. In one study on families with young children using only private well water, more educated mothers were more likely to treat their water [13]. A data gap exists regarding the characteristics of households that use these home water treatments, the frequency of use, and the efficacy of point-of-entry and point-of-use treatments for homes on private well and public water sources [12,14].

The objective of this study was to examine household water treatment in a cross-sectional sample of homes from a rural community in central Arizona that has a generally recognized arsenic contamination issue in both private well and municipal water sources [15]. We enumerate the types of treatments used in our study population, relate use of home water treatment to household demographic characteristics, summarize the contaminants measured in municipal and private well drinking water, and evaluate whether the treatments are effective at removing contaminants.

## 2. Materials and Methods

### 2.1 Sample Collection

In this study, we used data collected as part of a larger study: the Metals Exposure Study in Homes (MESH), which recruited 34 homes in a rural, central Arizona community to assess children’s exposures to metals in multiple environmental media. Enrolled households had at least one child between the ages of one to eleven years. Recruitment (door-to-door canvassing, mass mailings, advertisements in both pay for and free print media, and presence at local festivals) was performed between October 2011 and June 2013. Door-to-door recruitment and home visits to collect samples and administer questionnaires were completed by field technicians hired from the study community. Two home visits to each home were scheduled after enrollment. During the first visit, a home walk-through and questionnaires about basic home layout were conducted; during the second visit, approximately one to two weeks later, water and other environmental and biological samples were taken and questionnaires on demographics, home routines, child health, water treatment types, and water sources were administered. At least one untreated tap water sample was collected from each home following a two-minute flush. Bottled and hauled water sources were also recorded but were excluded from this analysis because water treatment data for those samples were unknown. Three of the 34 homes were not used in this analysis because they declined to provide information regarding home water treatment. This study was approved by the University of Arizona Human Subjects Protection Program.

### 2.2 Water Sample Analysis

Water samples were stored at 4 degrees Celsius and preserved within two weeks of collection with Optima trace metal free nitric acid to a pH<2. All samples were tested for turbidity, with a criterion of >1 Nephelometric Turbidity Unit (NTU) necessitating acid digestion prior to analysis. Turbidity levels for all samples were <1 NTU, so acid digestion was not performed. For purposes of quality control, duplicate samples were collected for approximately 10% of the homes, and samples were split in the laboratory for 6% of the homes. Relative percent differences were computed for both duplicates and splits to assess precision. Total metal(loid)s analysis was performed by the Arizona Laboratory for Emerging Contaminants using a Perkin Elmer ELAN DRC-II (for samples before April 2012) and an Agilent 7700× (after April 2012). Samples were also analyzed for the arsenic species, including arsenite (AsIII), aresenate (AsV), monomethylarsonic acid (MMA), dimethylarsinic acid (DMA), arsenobetaine (AsB), and arsenocholine (AsC) by ion chromatography coupled to inductively coupled plasma mass spectrometry [16]. USEPA Method 6020 [17] was followed for quality control/assurance procedures. Prior to metals analysis, calibration curves with at least five points and correlation coefficients >0.995 were created. An Initial Calibration Blank and Calibration Verification solution with a concentration in the low to mid-range of the calibration curve were analyzed. Each batch also included a quality control solution from a second source, such as the NIST 1643e Trace metals in water. The study limit of detection (LOD) was defined as three times the standard deviation of the blanks for each element in water. If the laboratory reported a value that was below the LOD, that value was used as the concentration. If the laboratory reported a non-detect or no value, this was replaced by the LOD divided by the square root of two [18]. Water hardness was calculated as mg/L of calcium carbonate (CaCO_3_) using the following equation [19]:

(1) $$\text{Hardness}(in\frac{mg}{L})\text{as CaC}O_{3}=M^{2+}(in\frac{mg}{L})\times \frac{50g/\text{equivalent}}{\text{EW of}M^{2+}}$$

where M^2+^ represents any divalent cation and EW represents the molecular weight of M^2+^ (g/mol) divided by the absolute value of the ion charge, which for the case of divalent cations is two. Major cations that contribute to water hardness include calcium, magnesium, strontium, iron, and manganese [19]. For our study, we only used magnesium and calcium to calculate hardness, because their concentrations were at least three orders of magnitude greater than those of other cations.

### 2.3 Data Analysis

Information on household habits, including water treatment and use, resident socio-demographics, and home characteristics were collected with a questionnaire administered in the participant’s home. Questionnaire responses were inputted into a database and checked for correctness and completeness in Microsoft Access 2010 (Microsoft, Redmond, WA). Simple exact logistic regression was used to determine if water source or any socio-demographic and home characteristics, including household income, years of education for adults, years lived in the home, home age, and number of children in the home could were associated with likelihood of home water treatment use (Table 3). To test the correlation among these variables, a Spearman correlation was run with Bonferroni correction of the p-values. To determine if contaminants concentrations in untreated tap water above MCL or USEPA National Secondary Drinking Water Regulation (NSDWR) guidelines was associated with likelihood of water treatment, we used simple exact logistic regression on log transformed contaminants concentrations and water hardness. All analyses were conducted in STATA/SE 13.1 (StataCorp, College Station, TX). An alpha level of 0.05 was used for statistical significance.

## 3. Results

### 3.1 Water Sources and Home Water Treatments

Of the 31 homes surveyed, 58% (n=18) were on private wells, 26% (n=8) were on a small municipal provider (serving 700 people), and 16% (n=5) were on a large municipal provider (serving 42,000 people) [5]. Home water treatments in the surveyed households included activated carbon (AC) filtration, reverse osmosis (RO), and water softener (WS) (Table 1). All AC and RO treatments were point-of-use units located immediately before tap discharge, and no AC treatments were pour-through pitcher types. All WS treatments were point-of-entry units, which treated water as it entered the home. Eight homes on municipal water and 10 homes on private well water reported no treatment. Of the 13 homes using water treatments, nine used only one treatment and four used multiple treatments (Table 1). The majority of homes on the small municipal provider used no home treatment, while the majority of homes on the large municipal provider used a home treatment. All homes using multiple treatments relied on private well water. Overall, the three treatment types were used equally, but frequency varied by water source. AC was the most common treatment for homes on municipal water, while homes on private well water used RO and WS more than AC.

### 3.2 Socio-demographic and Home Characteristics

Socio-demographic and home characteristics, including household income, years of education for adults, years lived in the home, home age, and number of children in the home, were compared by whether or not residents used home water treatment (Table 2). Notably, families who treat their water had an average income nearly $30,000 higher than those who did not treat. In addition, homes with treatment were, on average, over a decade newer than those without treatment. Simple exact logistic regression was used to determine if water source or any other variable was associated with likelihood of home water treatment use (Table 3). Significant predictors of water treatment included household income (OR = 1.25; p = 0.048) and average years of education for adults in the home (OR = 1.49; p = 0.003). Both were positively associated with home water treatment, while home age (OR = 0.94; p = 0.03) was the only significant inversely associated variable. To test the correlation among these variables, a Spearman correlation was run with Bonferroni correction of the p-values. Only the correlation between household income and years of education for adults approached significance (ρ= 0.47; p = 0.19), suggesting a moderate to strong but statistically insignificant relationship. Water source, years lived in the home, and number of children in the home were not associated with home water treatment.

### 3.3 Contaminants in Tap Water

**Figure 1:**
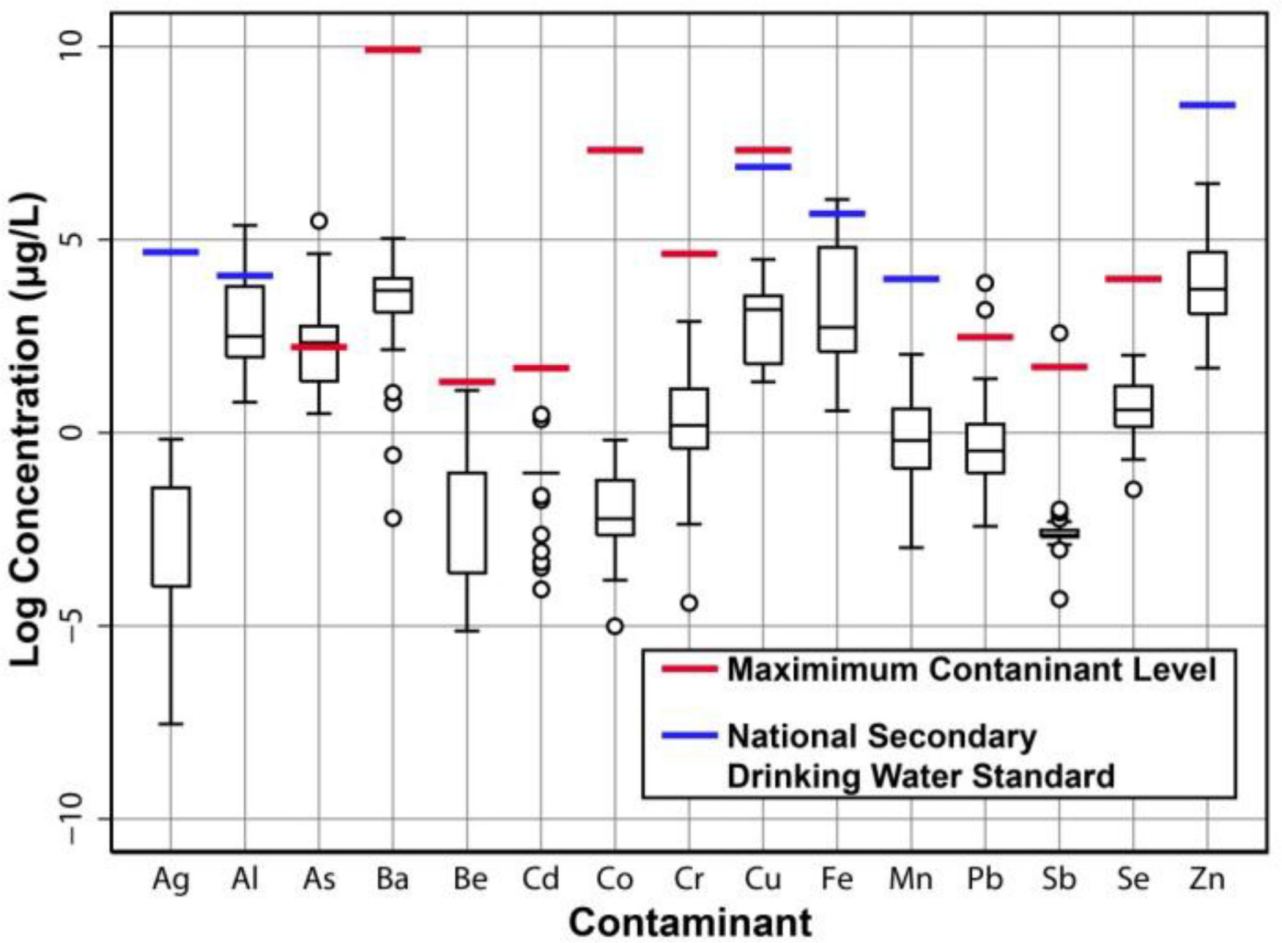
Distribution of contaminant levels in tap water samples without home treatment and their respective United States Environmental Protection Agency Maximum Contaminant Level (MCL) or National Secondary Drinking Water Regulation (NSDWR) guidelines (illustrated by red and blue solid lines, respectively). The median is represented by the line within the box; the 25th and 75th percentiles are represented by the lower and upper box bounds, respectively; Q1-1.5*(Q3-Q1) and Q3+1.5(Q3-Q1) are represented by the bottom and top ends of the whiskers, respectively. Note: all 34 homes are shown including the three excluded from analysis because of missing home treatment data.

Concentrations of contaminants in tap water samples without home treatments with their respective MCL or NSDWR guidelines are shown in Figure 1. Results are shown in this figure for all homes in the study, regardless of water source, an obvious influence on potential contaminant concentrations. Overall, few contaminant concentrations were above respective guidelines, however arsenic (As), lead (Pb), and antimony (Sb) levels exceeded the MCL in at least one home. Of note, nearly half of homes had water arsenic levels above the MCL; in that group, two homes had water arsenic levels >100 µg/L, more than 10 times the MCL. Two homes had lead concentrations above the MCL, of which one home had a concentration more than three times the MCL. Aluminum (Al) and iron (Fe) exceeded their respective NSDWR levels in at least one home. In addition, while there are no established health guidelines for water hardness for the US or the State of Arizona, very hard water (>300 mg/L as CaCO_3_) may promote scale deposition in piping and water heaters and poor tasting water, leading many consumers to soften water [20]. In our study, four homes had very hard water. Meanwhile, no homes in our study had untreated soft water (≤50 mg/L as CaCO_3_) [21], yet several homes’ RO treatments resulted in soft water. As such, we have included water hardness with contaminants that exceeded guidelines for analysis.

To determine if contaminant concentrations in untreated tap water above MCL or NSDWR guidelines were associated with likelihood of water treatment, we used simple exact logistic regression on log transformed concentrations and water hardness. However, the likelihood of home water treatment was not associated with concentrations of any contaminant or hardness levels (data not shown).

Notably, arsenic levels in untreated water samples exceeded the MCL in all homes on the small municipal supplier, while 37.5% homes on private wells and none of the homes on the large municipal supplier exceeded the arsenic MCL in untreated water (Table 4). Guideline exceedances for untreated water samples from private wells were also found in 31.3% of samples for aluminum and in 6.25% of samples for lead and antimony. Only one home on the small municipal water supplier was above guideline levels for aluminum or iron. The large municipal supplier had guideline exceedances in samples from two homes for aluminum and from one for iron. Lead and antimony concentrations each exceeded their respective MCLs in 6.25% of homes on private well water. In our study, four homes had very hard water (>300 mg/L as CaCO_3_), and no homes had soft water (≤50 mg/L as CaCO_3_).

### 3.4 Treatment Effectiveness

**Figure 2:**
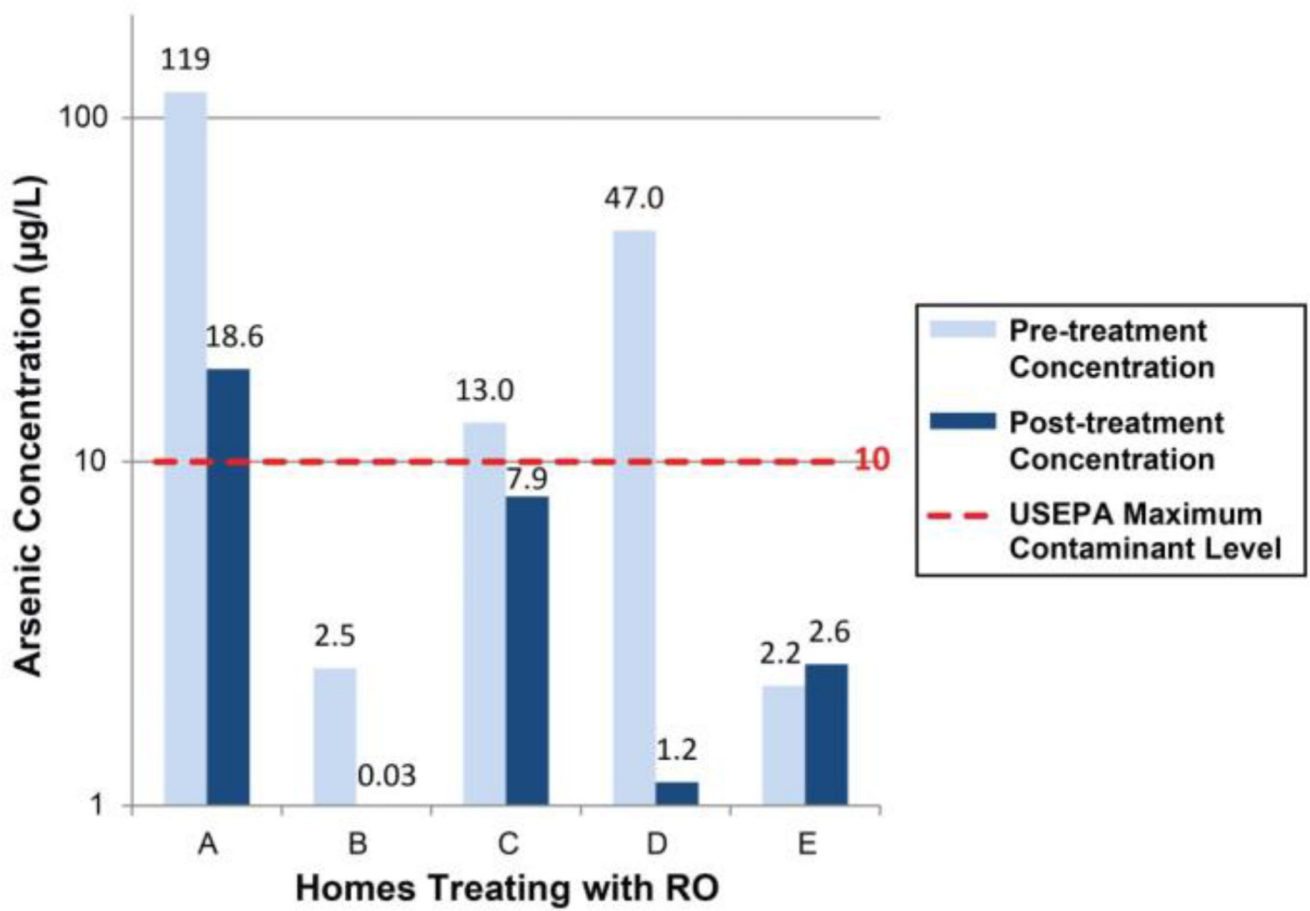
Arsenic concentrations in water samples before home treatment (Pre-treatment Concentration) and after RO treatment (Post-treatment Concentration) from homes with paired samples.

Various home treatments were effective in removing some contaminants and not others (Table 5). As expected, treatments often reduced the concentration of contaminants in the effluent relative to the influent water source, as indicated by ‘-’ concentration change (e.g. −51%). However, in some instances, the effluent concentration was higher than the influent concentration, as indicated by ‘+’ concentration change (e.g. +50%). Observations with limit of detection substitutions for both influent and effluent values were excluded. RO reduced arsenic levels by as much as 99%, while AC also reduced concentrations by as much as 45%. No treatment was consistently effective in reducing aluminum or iron below respective NSDWR guidelines, and effluent concentrations were greater than pre-treated levels for six homes. For lead, RO consistently reduced concentrations, with a median reduction of 61%, while AC offered inconsistent reductions. Using RO, one home reduced antimony levels by 78% to below the MCL, while AC offered minimal reductions. Water hardness levels were reduced 97% on average using RO. Unfortunately, due to the fact we could not access untreated water in many homes with WS, we were unable to test WS water hardness reduction efficiency. Focusing on the impacts of RO on arsenic concentration, Figure 2 shows arsenic concentrations in water samples before and after RO treatment in homes, illustrating the wide range of treatment efficiencies for RO.

## 4. Discussion

To our knowledge, this is the first study investigating home water treatments and their effectiveness in residences using both municipal and private well water sources in a cross-sectional sample of homes from a rural US community. In our study, 42% of homes treated their water with RO, AC, or WS. Independent of source water quality, residents of homes with higher household income or education levels were more likely to treat their water, while residents in older homes were less likely to do so. All homes on the small public water supply, no homes on the large public water supply, and 37% of homes on private wells had arsenic MCL exceedances. Home treatments had variable impacts, increasing some contaminant concentrations and decreasing others. These findings could help improve education and outreach on water testing and treatment, which may help reduce health risks from contaminated or improperly treated drinking water, especially in rural areas where residents more often rely on smaller municipal water supplies or unregulated private well water.

Five RO systems and five WS units were located in homes using private well water and four AC treatments were located in homes using municipal water. Such differences in treatment type among water sources suggest that the motivation for treating water varies by water source. The prevalence of RO and WS treatments among homes using private well water may indicate concern about reducing contaminants such as arsenic or water hardness. Meanwhile, the increased frequency of AC treatments in homes using municipal water suggests concern over undesirable tastes and odors. Unfortunately, we did not collect information on why residents do or do not treat their water and, if they do, their reason for choosing a particular treatment(s). A more thorough investigation of such information could lead to more germane education or outreach materials on the potential need and relevance of home water treatments.

Homes with higher household incomes or more years of education for adults in the home were significantly more likely to have home water treatment, independent of source water quality. In another study, household income was also found to be positively associated with treatment use [22]. Home treatment use has been both positively [13] and inversely [22] associated with maternal education, suggesting that how the level of education of adults is measured may impact the result. However, these other studies only investigated treatment habits of residents using private wells, while our study and others have shown that homes on small municipal providers may also need home water treatment [2–4]. In our study, owners of older homes were less likely to treat water, a hypothesis we believe has not been tested before. Water source, a variable not examined in other studies, was not significantly associated with home treatment, potentially suggesting a lack of consumer water quality knowledge. Interestingly, none of these variables were significantly correlated with each other, suggesting these factors are independently associated with home treatment. Nevertheless, our findings likely indicate that households with lower income levels are unable to afford home treatments even if they may want them, or they may choose to forego home treatment in favor of bottled water [6,12]. In addition, users must balance perceived risk and treatment effectiveness with cost and effort [2,12]. To remedy this potentially disproportionate impact on households that want home treatment but cannot afford it, implementing a subsidy program to help homes or smaller municipal water providers afford treatments could have a substantial impact on reducing contaminant exposure from drinking water in many communities [23].

There was no association between contaminant levels in untreated water and home treatment use, which may indicate residents are unaware of contaminant levels in their water, either because they did not test their well water or did not receive an annual water quality report from their municipal provider. However, it may also suggest that residents, even if they know contaminant concentrations in their water are above guidelines, believe their water is safe because there are no perceivable aesthetic water issues (i.e. odor, poor taste, etc.), no obvious health outcomes [24] such as development of disease, or that they simply do not view the contaminant level as an issue [25]. Future studies should inquire about residents’ water quality testing knowledge and experience, which may help predict whether or not homes would use treatment if they were aware of their water quality. In addition, this may also suggest a need for subsidized water testing and enhanced education about effects of consuming water with guideline exceedances in communities or areas with endemic high levels of contaminants, such as arsenic in our study region.

In our study, all untreated water samples in homes served by the small municipal supplier exceeded the MCL for arsenic, with some levels two times the MCL. This finding highlights a known concern: small water providers may be unable to provide drinking water within health-based guidelines [2–4]. At the time of this writing, the small municipal supplier in this community is working with the state environmental agency to install treatment to reduce arsenic in their supply [26]. While guideline exceedances are equally important to homes on private well and public water supplies, these exceedances in public water supplies are particularly concerning given that, unlike private well owners who are responsible for their own water testing and treatment, residents served by public providers expect contaminant levels to be below guidelines without having to test for contaminants such as arsenic, let alone treat for them. Furthermore, the fact that all sampled homes served by the small municipal provider had arsenic MCL violations is especially distressing given the myriad health effects of arsenic [6,27–29]. As such, this and similar situations necessitate sustained outreach and education about water testing and treatment for homes served by private wells and small municipal providers, especially in areas of endemic elevated groundwater contaminants.

In homes using treatments, some contaminants were reduced while others were not. RO effectively decreased concentrations of lead, antimony, and arsenic. Notably, of three homes treating with RO (homes A, C, and D), two of them reduced arsenic levels below the MCL (C and D) (Figure 2). In our speciated analysis, we only detected arsenate (AsV), for which RO is the recommended treatment [30]. It is possible that non-ionic particulate species, which are less effectively removed by RO treatment [30], may have been present in our water [16]. If so, this may explain the range of removal efficiencies, however, this is unlikely due to the very low turbidity in all samples. RO efficiencies may also be reduced with age and condition of the membrane, pH, and CaCO_3_ precipitation potential [31], however, we did not record the information needed to assess these. It is important to note, that for homes like home A in Figure 2, even though RO removed 85% of arsenic, the effluent concentration was still above the MCL due to the high initial concentration [9,23]. This point would be crucial for improved education campaigns on home treatments for arsenic, as homes with such high arsenic levels have limited options: treat RO-treated water with an additional contaminant reduction method or use another water source, such as bottled or hauled water.

RO treatment also reduced water hardness to 3.30 mg/L as CaCO_3_ (soft water) in two homes. While very hard water (>300 mg/l as CaCO_3_) may lead to scaling on pipes and water heaters and poor taste [20,32], water that is too soft (≤50 mg/L CaCO_3_) may not benefit cardiovascular and skeletal health and guard against certain cancers, as has been shown in consumers drinking harder water [32–34]. Additionally, soft water may corrode piping systems, potentially leaching iron or lead and increasing concentrations of these metals in water [32,35]. This may explain why in the two aforementioned homes using RO, arsenic and hardness decreased by 99% and 97%, respectively, while yet iron increased by 98% in one, while in the other, arsenic, hardness, and iron decreased by 84%, 97%, and 5%, respectively. Residents in homes with very soft water may consider remineralizing their water to prevent corrosion [35] or supplementing their magnesium or calcium intake [20,32,33]. AC had mixed reduction efficiencies and often had no effect or increased concentrations in effluent. Though no AC effluent levels were above health-based guidelines, this suggests that consumers may be unaware of the range of complex effects treatments may have on their water quality. Increased education on how water treatments may both increase and decrease chemical concentrations, as well as the need to test both influent and effluent waters, may help consumers choose and properly maintain the most advantageous treatment(s) for their water quality.

In conclusion, we found that about half the homes in the study used RO, AC, or WS treatment. We discovered that residents with increased household income and education levels were more likely to use home treatment, while residents in older homes were less likely to do so. For arsenic, all homes on the small public water supply, 33% of homes on private wells, and none of the homes on the large public water supply had MCL exceedances. Home treatments both increased and decreased contaminant levels. To reduce the public health burden from untreated or improperly treated drinking water, we recommend increasing home water testing and treatment education for residents on unregulated private wells, as well as those on smaller municipal suppliers. In addition, subsidies to defray the cost of water testing and home treatment installation and maintenance may help poorer families obtain treatment systems they may want but are unable to afford. Both campaigns would be especially important in areas with lower income and education levels and with endemic water contamination issues, such as arsenic in the southwestern US. Through these interventions, consumers would be better equipped to decide if their water required treatment and what treatment would be appropriate to reduce contaminants. In doing so, resources could be more effectively directed to communities most in need, resulting in a greater public health impact while reducing rural health disparities.

## Figures and Tables

**Table 1:** Number of homes using various home water treatments (n=31). RO: reverse osmosis, AC: activated carbon, and WS: water softener.

Water Source				
Treatment	Small Municipal (n=8, 25.8%)	Large Municipal (n=5, 16.1%)	Private Well (n=18, 58.1%)	Total
No treatment	6 (75.0%)	2 (40.0%)	10 (55.6%)	18 (58.1%)
1 Treatment: RO	1 (12.5%)	0 (0%)	1 (5.56%)	2 (6.45%)
1 Treatment: AC	1 (12.5%)	3 (60.0%)	1 (5.56%)	5 (16.1%)
1 Treatment: WS	0 (0%)	0 (0%)	2 (11.1%)	2 (6.45%)
>1 Treatment: RO, AC	0 (0%)	0 (0%)	1 (5.56%)	1 (3.22%)
>1 Treatment: RO, WS	0 (0%)	0 (0%)	3 (16.7%)	3 (9.68%)

**Table 2:** Summary statistics for socio-demographic and home characteristics by home treatment. n: number of homes in treatment group, GM: geometric mean, and GSD: geometric standard deviation.

Variable	Treatment	n	Mean	GM	GSD
Household Income ($)	Yes	12	96,700	91,700	14,200
	No	16	67,100	55,600	19,000
Average Years of Education for Adults in the Home	Yes	13	18	18	1
	No	18	15	15	1
Years Lived in the Home	Yes	13	5.5	4.2	2.5
	No	17	5.6	2.1	9.1
Home Age (Years)	Yes	13	13.8	9.1	2.8
	No	18	25.3	20.3	2.1
Number of Children in Home	Yes	13	3	3	2
	No	18	3	3	2

**Table 3:** Results of simple exact logistic regression analyses assessing tap water treatment (yes or no) according to water source and socio-demographic and home characteristics.

Variables	Odds Ratio	95%CI
Household Income	1.25	1.00 – 1.64*
Average Years of Education for Adults in the Home	1.49	1.12 – 2.12**
Years Lived in the Home	1.00	0.83 – 1.20
Home Age (Years)	0.94	0.89 – 0.99*
Number of Children in Home	1.16	0.70 – 2.02
Water Source		
Private Well (Ref)		
Municipal	0.78	0.18 – 3.34
Detailed Water Source		
Private Well (Ref)		
Small Municipal	0.42	0.07 – 2.65
Large Municipal	1.88	0.25 – 14.1

Note:

* p-value<0.05;

** p-value<0.01.

**Table 4:** Contaminant and water hardness concentrations in untreated water by source and number of homes exceeding either USEPA Maximum Contaminant Level (MCL) or National Secondary Drinking Water Regulation (NSDWR) guidelines. Note: Percentages are based on total homes on that water source with a contaminant measurement (n).

			Concentration (µg/L)*			

Contaminant (Guideline)	Water Source	n	Min	Median	Max	Homes Exceeding Guidelines
Aluminum (NSDWR: 50 µg/L)	Small Municipal	8	2.21	7.12	96.0	1 (12.5%)
	Large Municipal	5	7.10	30.8	215	2 (40.0%)
	Private Well	16	2.93	22.1	153	5 (31.3%)
Arsenic (MCL: 10 µg/L)	Small Municipal	8	10.3	11.4	19.0	8 (100%)
	Large Municipal	5	2.84	3.97	5.06	0 (0%)
	Private Well	16	1.84	6.17	251	6 (37.5%)
Iron (NSDWR: 300 µg/L)	Small Municipal	7	6.65	41.8	421	1 (14.3%)
	Large Municipal	5	8.19	17.5	389	1 (20.0%)
	Private Well	16	2.17	14.6	334	1 (6.25%)
Lead (MCL: 15 µg/L)	Small Municipal	8	0.39	0.60	0.87	0 (0%)
	Large Municipal	5	0.25	1.25	12.4	0 (0%)
	Private Well	16	0.15	0.65	48.1	1 (6.25%)
Antimony (MCL: 6 µg/L)	Small Municipal	7	0.04	0.07	0.14	0 (0%)
	Large Municipal	5	0.01	0.05	0.07	0 (0%)
	Private Well	16	0.06	0.07	11.0	1 (6.25%)
Water Hardness*	Small Municipal	7	76.2	132	157	
	Large Municipal	5	99.9	119	128	
	Private Well	11	74.4	267	605	

Note:

* Water hardness values in mg/L.

**Table 5:** Percent change in concentration for contaminants with concentrations exceeding either MCL or NSDWR guidelines and for water hardness by treatment type (RO: reverse osmosis; AC: activated carbon; WS: water softener). Increases of contaminant concentration in effluent relative to influent are denoted with a ‘+’; decreases of contaminant concentration in effluent relative to influent are denoted with a ‘−‘. Note, N/A: not applicable.

			Concentration Change		

Contaminant (Guideline)	Treatment	n	Max Increase	Median	Max Decrease
Aluminum (NSDWR: 50 µg/L)	RO	5	+50%	+8%	−51%
	AC	5	+99%	+10%	−85%
	WS	1		+99%	
Arsenic (MCL: 10 µg/L)	RO	5	+15%	−81%	−99%
	AC	5	N/A	−24%	−45%
	WS	1		−20%	
Iron (NSDWR: 300 µg/L)	RO	3	+98%	−3%	−35%
	AC	5	+51%	0%	−77%
Lead (MCL: 15 µg/L)	RO	4	N/A	−61%	−90%
	AC	4	+16%	0%	−31%
	WS	1		−71%	
Antimony (MCL: 6 µg/L)	RO	1		−78%	
	AC	2	N/A	−5%	−11%
Water Hardness	RO	2	N/A	−97%	−97%
	AC	2	+5%	−1%	−8%
